# World Malaria Day 2016 in the Kingdom of Cambodia: high-level governmental support embodies the WHO call for “political will to end malaria”

**DOI:** 10.1186/s12936-016-1359-6

**Published:** 2016-06-02

**Authors:** Sara E. Canavati, Cesia E. Quintero, Thavrin Bou, Virak Khieu, Rithea Leang, Dysoley Lek, Po Ly, Sinuon Muth, Kim Seng Lim, Luciano Tuseo, Sovann Yok, Kunthearith Yung, Jack S. Richards, Huy Rekol

**Affiliations:** Centre for Biomedical Research, Burnet Institute, Melbourne, Australia; Department of Clinical Tropical Medicine, Faculty of Tropical Medicine, Mahidol University 420/6 Ratchawithi Road, Bangkok, 10400 Ratchathewi Thailand; The National Center for Parasitology, Entomology and Malaria Control, Ministry of Health, Corner street 92, Trapaing Svay Village, Sankat Phnom Penh Thmey, Khan Sensok, Phnom Penh, Cambodia; Malaria Consortium Cambodia, Phnom Penh Office, House #91, St. 95, Boeung Trabek, Chamcar Morn, Phnom Penh, Cambodia; WHO Representative Office in Cambodia, # 61-64 Norodam Preah Blvd, Penh Phnom Penh, Cambodia; Provincial Malaria Supervisor, Provincial Health Department, Pailin City, Pailin Province Cambodia; School Health Department, Youth and Sports, Ministry of Education, Phnom Penh, Cambodia

**Keywords:** Malaria elimination, World Malaria Day, Cambodia, Political will

## Abstract

On World Malaria Day 2016, The Kingdom of Cambodia’s National celebrations served as a prime of example of how political will is currently being exercised in Cambodia through high-level governmental support for malaria elimination. The main country event was well-planned and coordinated by the National Programme for Parasitology, Entomology and Malaria Control (CNM), and included key contributions from high-ranking political figures, such as His Excellency (H.E) Mam Bun Heng (Minister of Health), and H.E. Keut Sothea (Governor of Pailin Province). There were more than 1000 attendees, ranging from Village Malaria Workers and high school students to CNM’s director and other officials in Pailin Province, Western Cambodia. A strong inter-sectoral participation included attendances from the Ministry of Education and high-level representatives of the Cambodian Armed Forces, as well as Malaria Partners like the World Health Organization.

## Background

Over the last decade, the Kingdom of Cambodia has made significant gains in the fight against malaria [1]. It is now working towards the ambitious target of eliminating *Plasmodium falciparum* malaria by 2030. Nevertheless, maintaining gains and reaching this goal will require a sustained, concerted effort by all sectors, including the highest levels of government. The World Health Organization (WHO) has recently advised that “through […] political will, affected countries can speed progress towards malaria elimination and contribute to the broader development agenda as laid out in the 2030 Agenda for Sustainable Development” [2]. On World Malaria Day 2016, National Celebration in The Kingdom of Cambodia served as a prime of example of how political will is currently being exercised in Cambodia through high-level governmental support for malaria elimination.

## Overview

This year on World Malaria Day the WHO, National Malaria Programmes and Malaria Partners rallied around the theme *End malaria for good*, which “reflects the vision of a malaria-free world set out in the WHO Global technical strategy for malaria 2016–2030” [3]. Cambodia’s World Malaria Day celebrations, coordinated by the National Programme for Parasitology, Entomology and Malaria Control (CNM), were held on Monday April 25th in Pailin Province, Kingdom of Cambodia. Most other provinces also held celebrations on the day. Strong political will to achieve malaria elimination in Cambodia was palpable at the highest levels of government. The event was hosted by the Provincial Health Department of Pailin Province and the Provincial Malaria Supervisor from Pailin Province, Dr. Yok Sovann. There was extensive provincial and national media coverage of the event.

## Opening ceremony

**Fig. 1:**
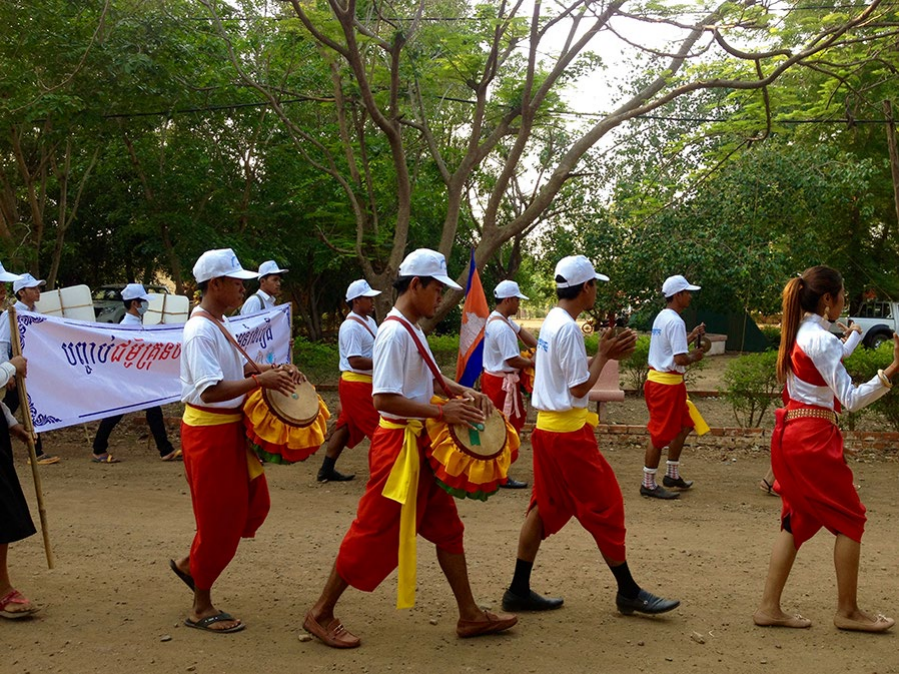
Performances by school students and music by a Khmer cultural band

**Fig. 2:**
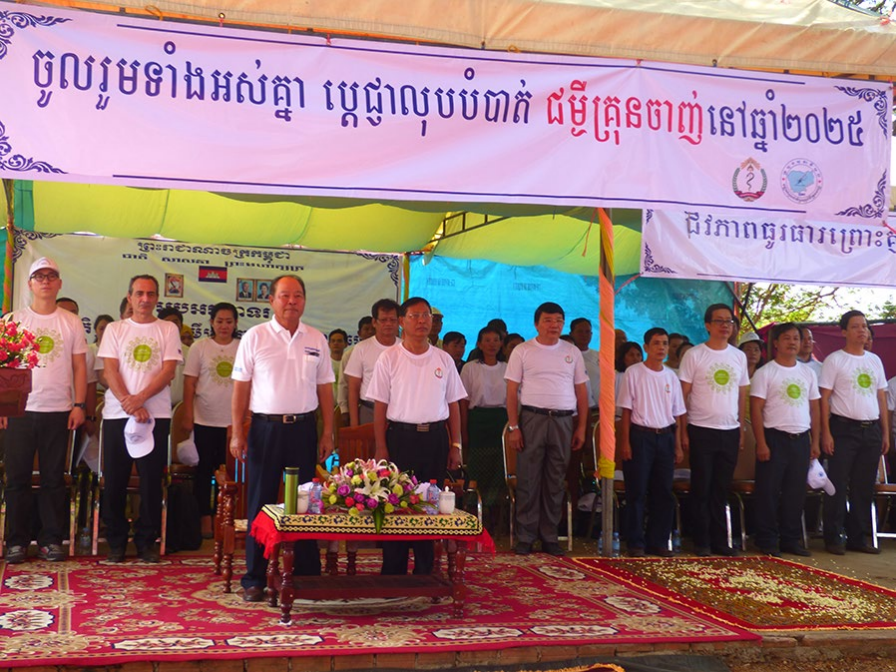
Speeches by the Minister of Health, His Excellency (H.E) Mam Bun Heng, H.E. Keut Sothea the Governor of Pailin Province, and Dr. Luciano Tuseo, Head of Malaria Programme, WHO Representative Office in Cambodia

The colourful opening ceremony took place in the courtyard of Hun Sen Krong Tep Nimith Pailin High School, and included performances by school students and music by a Khmer cultural band (Fig. 1). All health partners were present to welcome the Minister of Health, His Excellency (H.E) Mam Bun Heng, and the Governor of Pailin Province, H.E. Keut Sothea, both of whom showed their support for health strengthening efforts in the Kingdom of Cambodia earlier in the day by visiting the Antenatal Clinic for Mothers and Babies at the Pailin Referral Hospital. Speeches by H.E. Mam Bun Heng, H.E. Keut Sothea, and Dr. Luciano Tuseo (Head of Malaria Programme, WHO Representative Office in Cambodia, took the opportunity to celebrate the significant advances in malaria control that have led to a major decline in malaria morbidity and mortality, both globally and within the Kingdom of Cambodia, in recent years (Fig. 2).

The event had more than 1000 attendees, which also included the Governor of Pailin Province, Dr. Rekol Huy (Director, CNM), CNM Deputy Director and Vice-Director were in attendance, as well as the directors of various units within CNM, including the *Neglected Tropical Diseases Unit*, the *Health Education Unit*, the *Village Malaria Worker Unit* and the *Health Research Unit* and the *National Dengue Control Programme*. Representatives of other National Institutes of Health, including the Ministry of Health’s *Health System Strengthening Unit* were also present. A strong inter-sectoral participation included attendances from the School Health Department, the Ministry of Education’s Youth and Sports Division, and High-level Representatives of the Cambodian Armed Forces. Village Malaria Workers were also present at the event. Malaria Partners in attendance included the WHO, The United Nations Office for project services, The Burnet Institute, The Malaria Consortium, Health Poverty Action, and Population Services Khmer. The Malaria Partners pledged to intensify technical and coordination efforts in order to support the CNM in its fight against malaria.

The stakeholders also highlighted that significant work remains to be done, as approximately 3.2 billion people—nearly half of the world’s population—are still at risk of malaria, and millions are unable to access malaria prevention and treatment services. They detailed the World Health Assembly’s ambitious goal of reducing malaria morbidity and mortality by at least 90 %, and eliminating malaria in at least 35 countries, over the next 15 years; reaching this goal will require financial investments in malaria elimination to triple by 2030 [4]. It was emphasized that effective programme management, improved implementation of behaviour change communication, and intensified surveillance systems, must all be implemented in order to meet elimination targets in the Kingdom of Cambodia [5].

## Activities

**Fig. 3:**
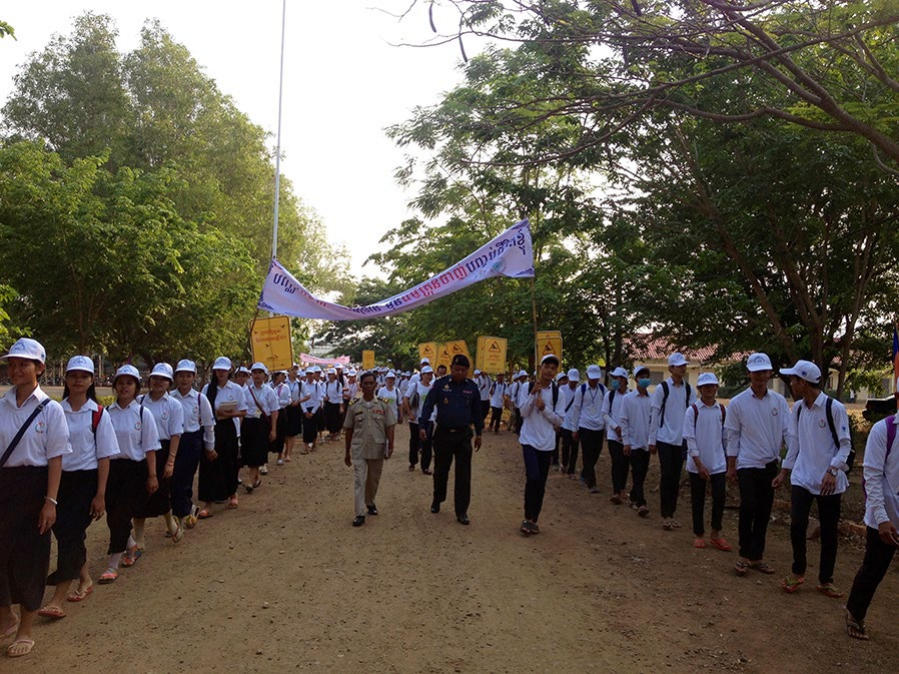
Students holding numerous malaria awareness signs

**Fig. 4:**
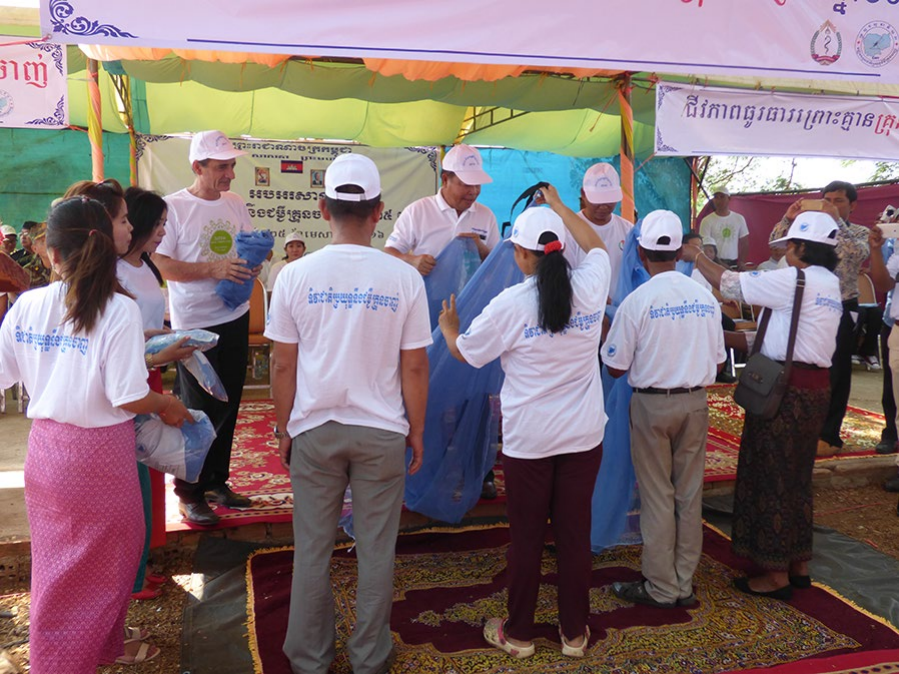
H.E. Mam Bun Heng emphasized the importance of using LLINs, and demonstrated their use among village malaria workers

**Fig. 5:**
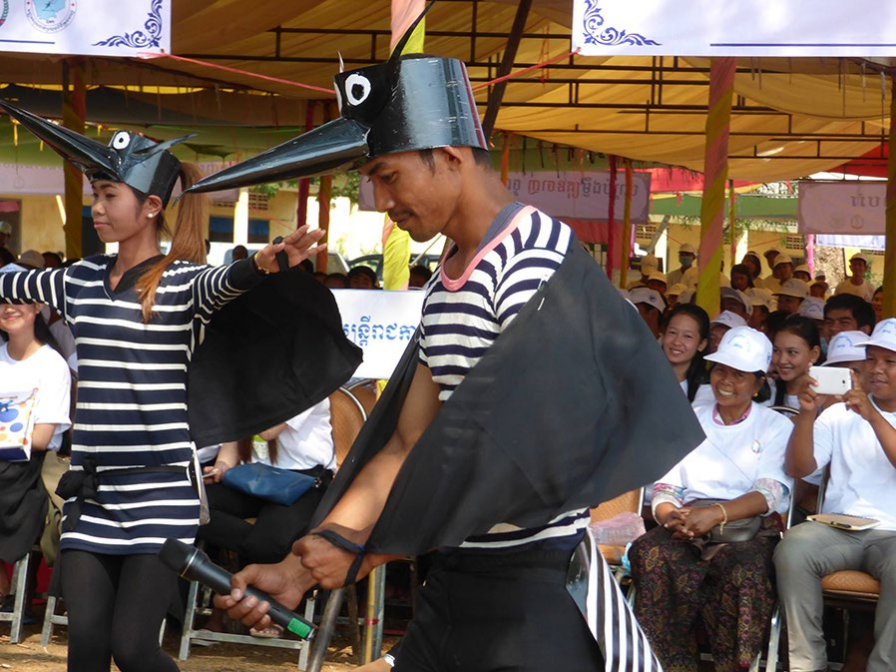
The mosquito costumes used in malaria skit (detail)

**Fig. 6:**
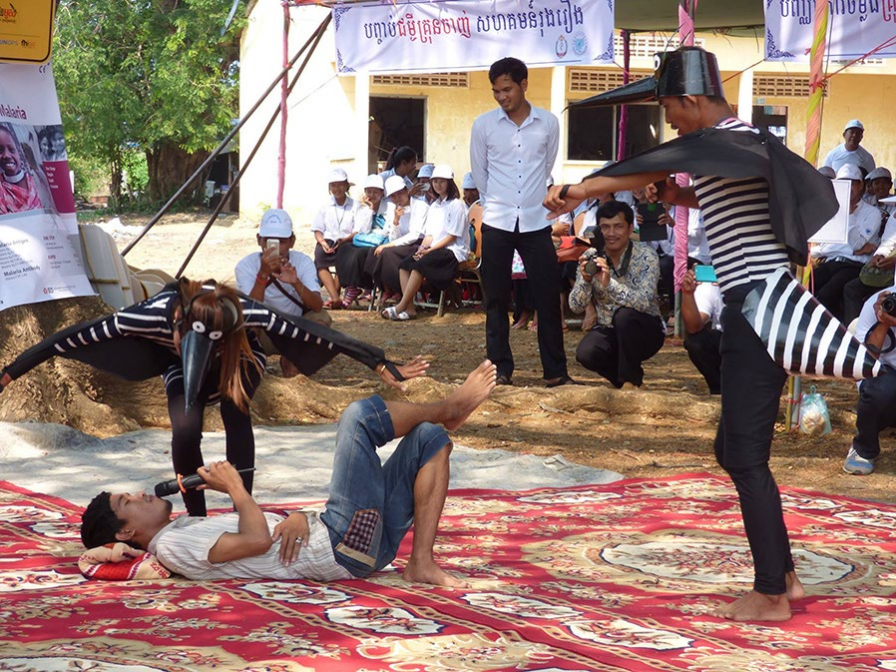
The mosquito costumes used in malaria skit

After the opening ceremony, several activities took place, including a cultural Khmer procession, and the distribution of T-shirts and hats bearing the logo “End malaria for good”. There were booth displays by CNM and NGOs presenting their work on behaviour change communication, health education, rapid diagnostic tests, and long-lasting insecticide-treated nets. Students held numerous awareness signs (Fig. 3), and were awarded prizes for participating in a quiz on malaria prevention and control administered by teachers. A skit about a man who contracts malaria by sleeping in the forest without a mosquito net, and is subsequently treated by a Village Malaria Worker, was performed by high school students. H.E. Mam Bun Heng emphasized the importance of using insecticide-treated nets, and demonstrated their use among village malaria workers (Fig. 4). The mosquito costumes were rated by event participants as some of the best ever from World Malaria Day events around the world (Figs. 5, 6).

## Conclusions

Despite its other pressing health needs, the Kingdom of Cambodia has been able to keep malaria elimination at the top of their agenda. On World Malaria Day, this could be seen not only through the CNM’s strong leadership and coordination efforts that led to a successful celebration, but also through the extent of inter-sectorial collaborations and the presence of high-ranking political figures who lent their time and support to the cause of malaria elimination.
